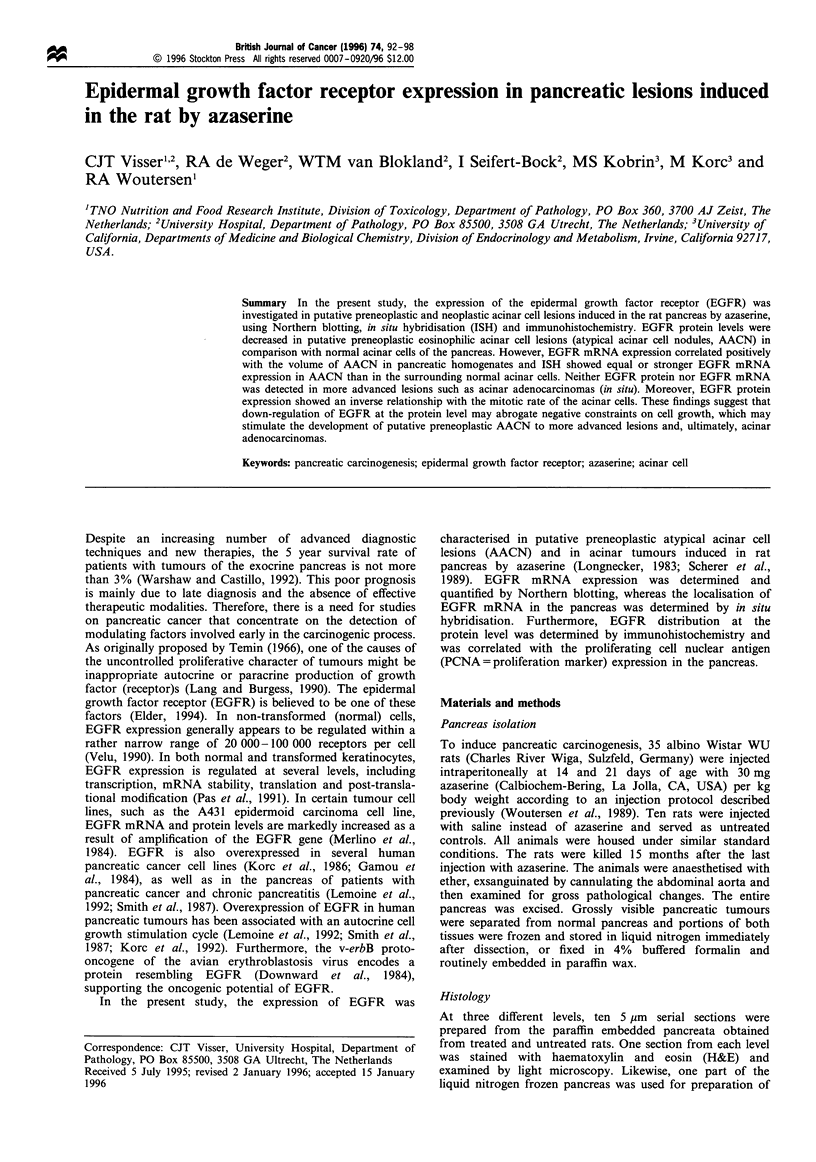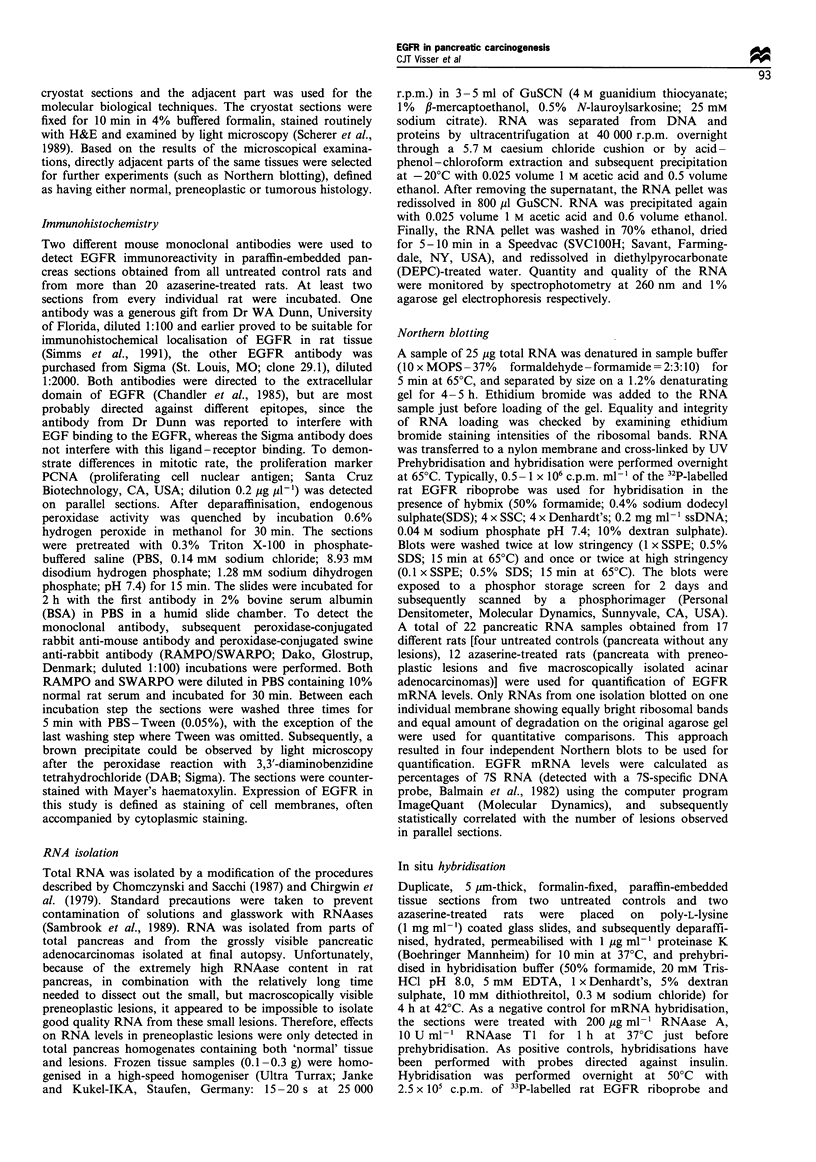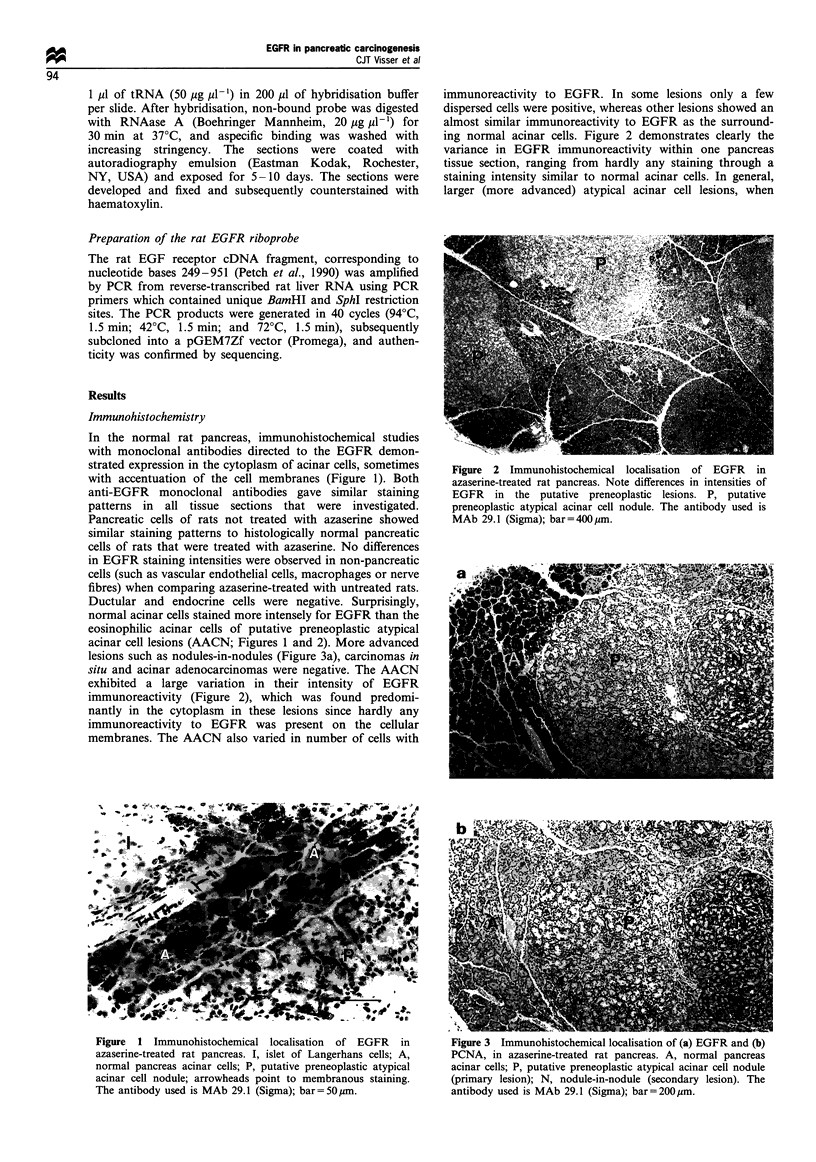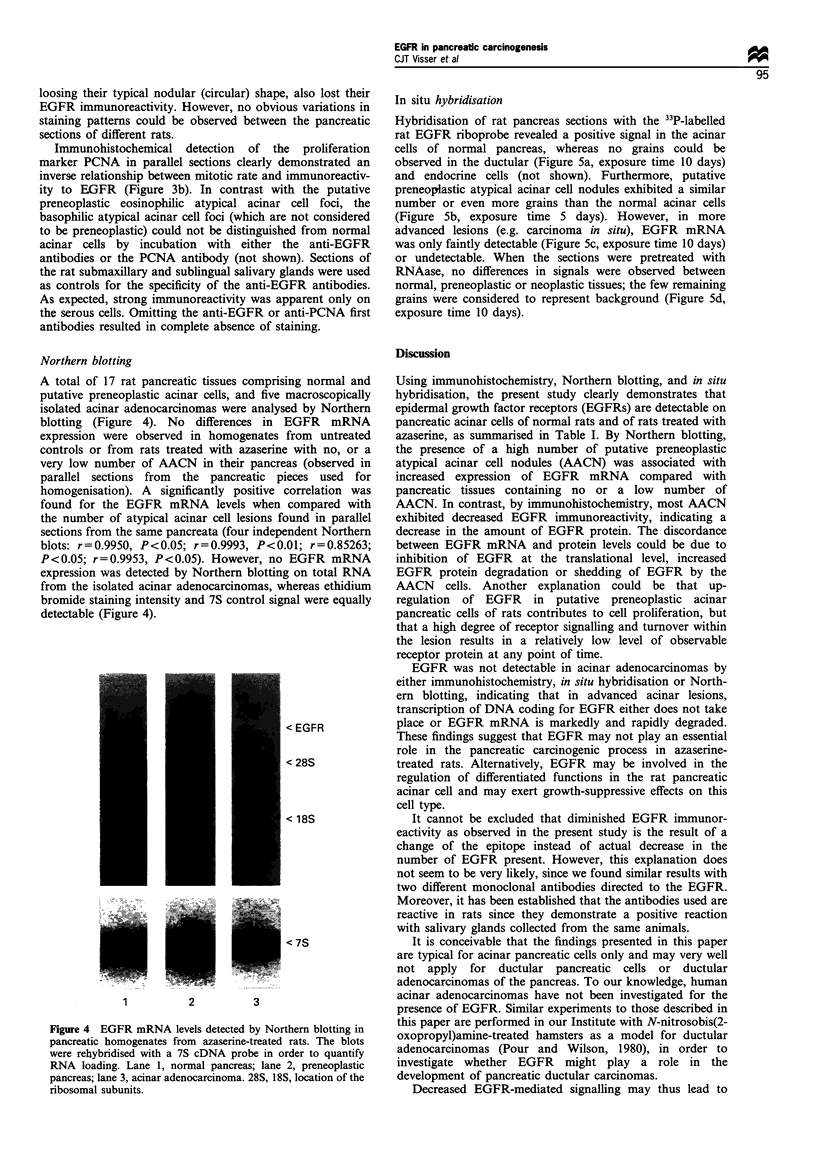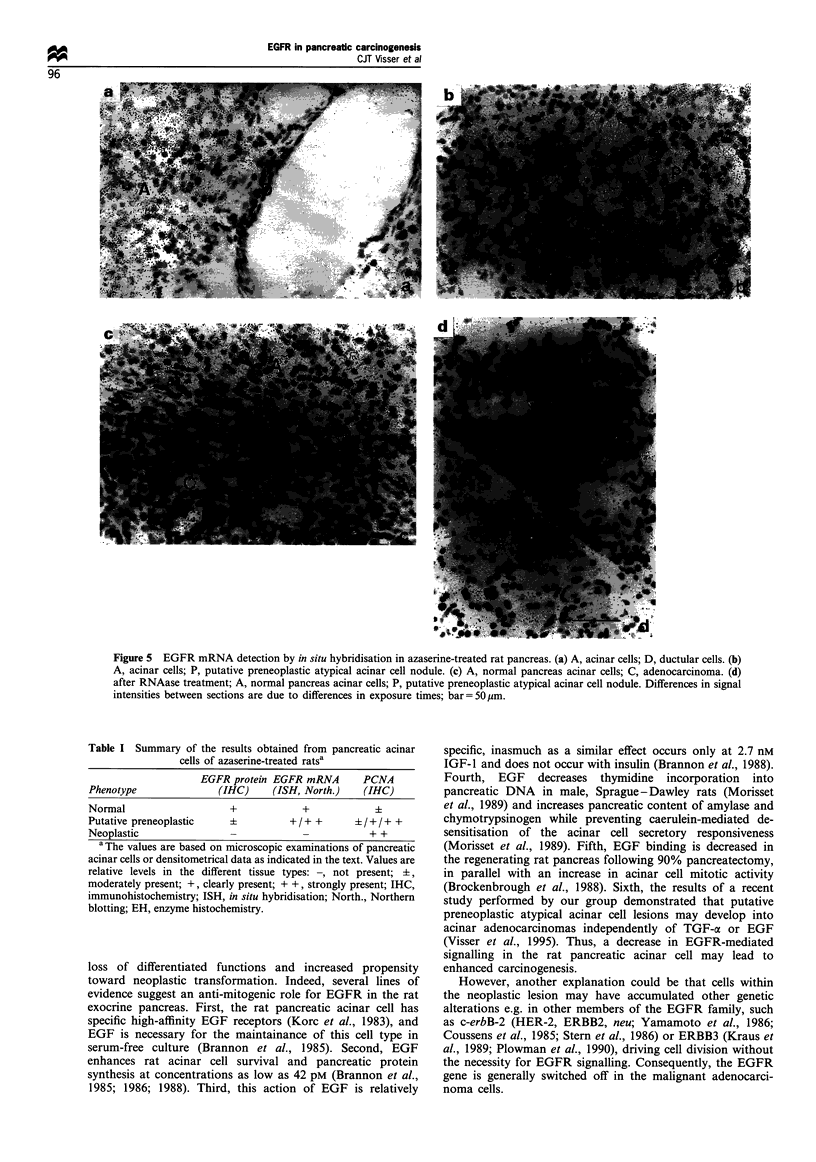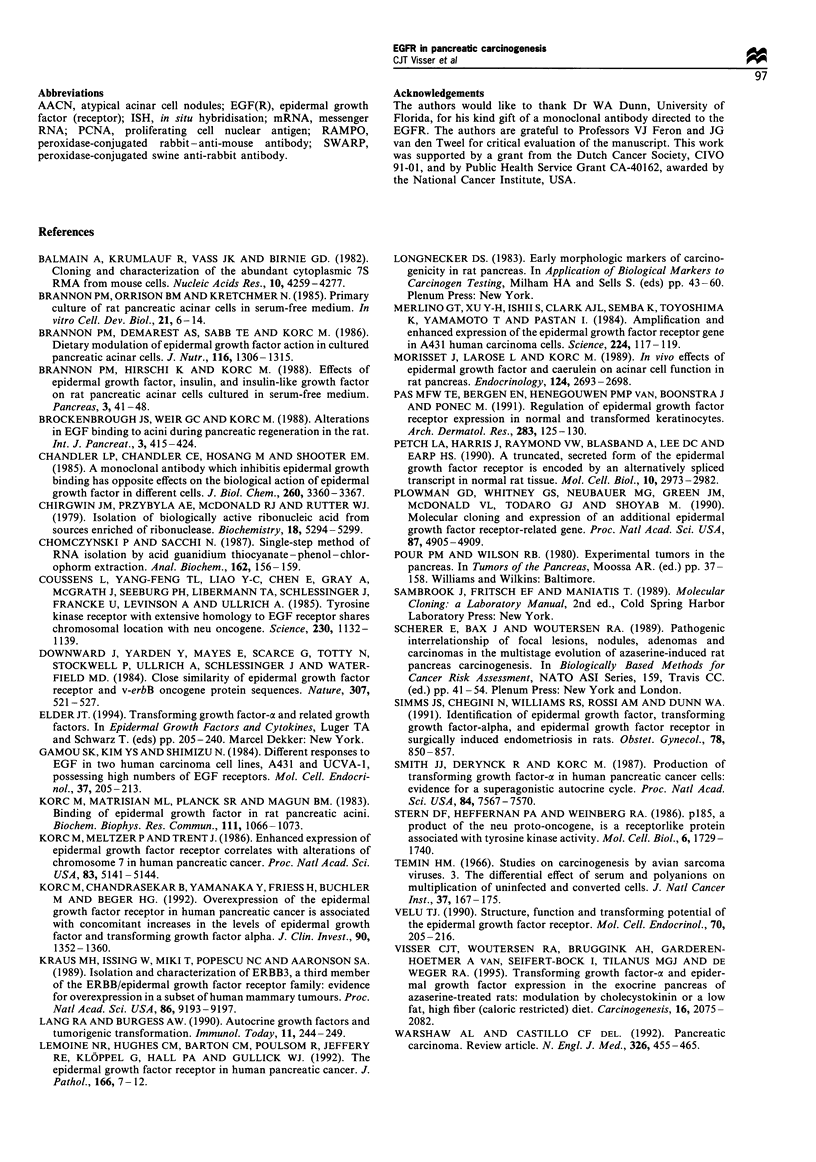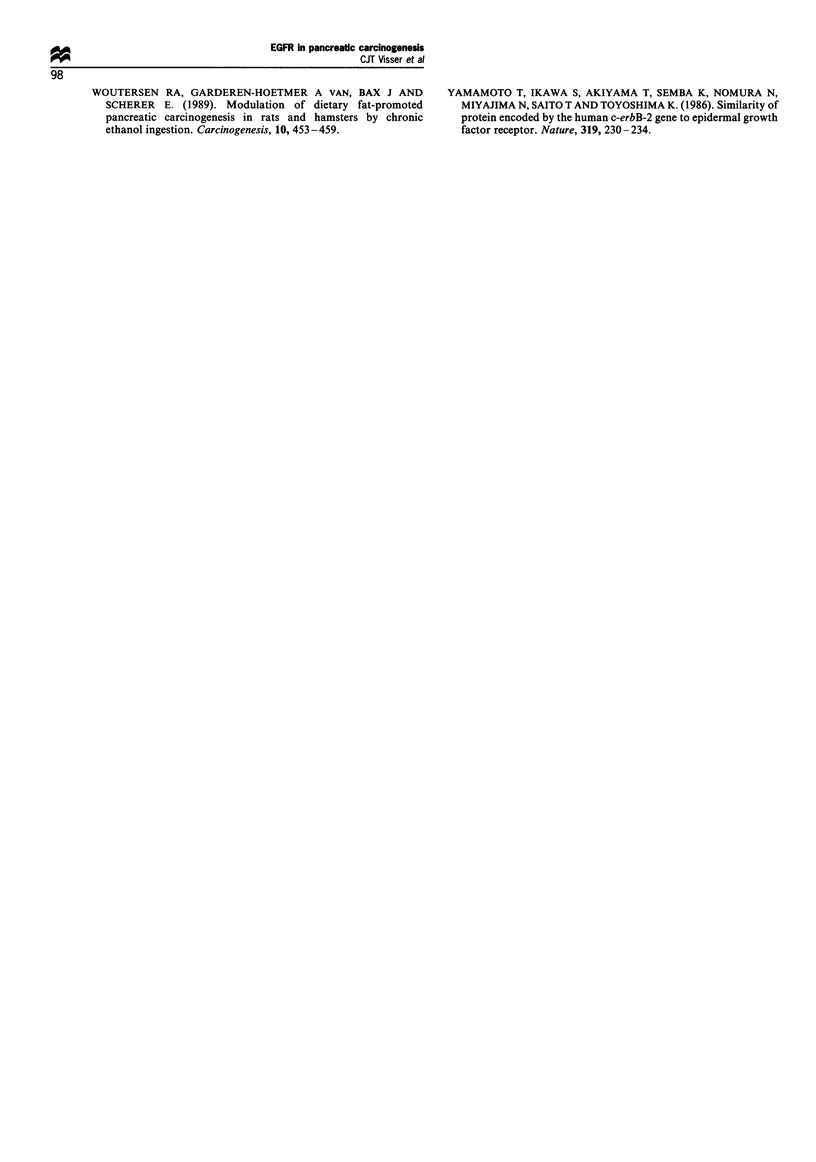# Epidermal growth factor receptor expression in pancreatic lesions induced in the rat by azaserine.

**DOI:** 10.1038/bjc.1996.321

**Published:** 1996-07

**Authors:** C. J. Visser, R. A. de Weger, W. T. van Blokland, I. Seifert-Bock, M. S. Kobrin, M. Korc, R. A. Woutersen

**Affiliations:** TNO Nutrition and Food Research Institute, Department of Pathology, Zeist, The Netherlands.

## Abstract

**Images:**


British Journal of Cancer (1996) 74, 92-98
© 1996 Stockton Press All rights reserved 0007-0920/96 $12.00

### Epidermal growth factor receptor expression in pancreatic lesions induced in the rat by azaserine

CJT Visser[1,2], RA de Weger[2], WTM van Blokland[2], I Seifert-Bock[2], MS Kobrin[3], M Korc[3] and RA Woutersen[1]

[1]*TNO Nutrition and Food Research Institute, Division of Toxicology, Department of Pathology, PO Box 360, 3700 AJ Zeist, The Netherlands;* [2]*University Hospital, Department of Pathology, PO Box 85500, 3508 GA Utrecht, The Netherlands;* [3]*University of California, Departments of Medicine and Biological Chemistry, Division of Endocrinology and Metabolism, Irvine, California 92717, USA.*

**Summary** In the present study, the expression of the epidermal growth factor receptor (EGFR) was investigated in putative preneoplastic and neoplastic acinar cell lesions induced in the rat pancreas by azaserine, using Northern blotting, *in situ* hybridisation (ISH) and immunohistochemistry. EGFR protein levels were decreased in putative preneoplastic eosinophilic acinar cell lesions (atypical acinar cell nodules, AACN) in comparison with normal acinar cells of the pancreas. However, EGFR mRNA expression correlated positively with the volume of AACN in pancreatic homogenates and ISH showed equal or stronger EGFR mRNA expression in AACN than in the surrounding normal acinar cells. Neither EGFR protein nor EGFR mRNA was detected in more advanced lesions such as acinar adenocarcinomas (*in situ*). Moreover, EGFR protein expression showed an inverse relationship with the mitotic rate of the acinar cells. These findings suggest that down-regulation of EGFR at the protein level may abrogate negative constraints on cell growth, which may stimulate the development of putative preneoplastic AACN to more advanced lesions and, ultimately, acinar adenocarcinomas.

**Keywords:** pancreatic carcinogenesis; epidermal growth factor receptor; azaserine; acinar cell

Despite an increasing number of advanced diagnostic techniques and new therapies, the 5 year survival rate of patients with tumours of the exocrine pancreas is not more than 3% (Warshaw and Castillo, 1992). This poor prognosis is mainly due to late diagnosis and the absence of effective therapeutic modalities. Therefore, there is a need for studies on pancreatic cancer that concentrate on the detection of modulating factors involved early in the carcinogenic process. As originally proposed by Temin (1966), one of the causes of the uncontrolled proliferative character of tumours might be inappropriate autocrine or paracrine production of growth factor (receptor)s (Lang and Burgess, 1990). The epidermal growth factor receptor (EGFR) is believed to be one of these factors (Elder, 1994). In non-transformed (normal) cells, EGFR expression generally appears to be regulated within a rather narrow range of 20 000-100 000 receptors per cell (Velu, 1990). In both normal and transformed keratinocytes, EGFR expression is regulated at several levels, including transcription, mRNA stability, translation and post-translational modification (Pas *et al.*, 1991). In certain tumour cell lines, such as the A431 epidermoid carcinoma cell line, EGFR mRNA and protein levels are markedly increased as a result of amplification of the EGFR gene (Merlino *et al.*, 1984). EGFR is also overexpressed in several human pancreatic cancer cell lines (Korc *et al.*, 1986; Gamou *et al.*, 1984), as well as in the pancreas of patients with pancreatic cancer and chronic pancreatitis (Lemoine *et al.*, 1992; Smith *et al.*, 1987). Overexpression of EGFR in human pancreatic tumours has been associated with an autocrine cell growth stimulation cycle (Lemoine *et al.*, 1992; Smith *et al.*, 1987; Korc *et al.*, 1992). Furthermore, the v-*erb*B proto-oncogene of the avian erythroblastosis virus encodes a protein resembling EGFR (Downward *et al.*, 1984), supporting the oncogenic potential of EGFR.

In the present study, the expression of EGFR was characterised in putative preneoplastic atypical acinar cell lesions (AACN) and in acinar tumours induced in rat pancreas by azaserine (Longnecker, 1983; Scherer *et al.*, 1989). EGFR mRNA expression was determined and quantified by Northern blotting, whereas the localisation of EGFR mRNA in the pancreas was determined by *in situ* hybridisation. Furthermore, EGFR distribution at the protein level was determined by immunohistochemistry and was correlated with the proliferating cell nuclear antigen (PCNA = proliferation marker) expression in the pancreas.

Correspondence: CJT Visser, University Hospital, Department of Pathology, PO Box 85500, 3508 GA Ultrecht, The Netherlands
Received 5 July 1995; revised 2 January 1996; accepted 15 January 1996

### Materials and methods

### *Pancreas isolation*

To induce pancreatic carcinogenesis, 35 albino Wistar WU rats (Charles River Wiga, Sulzfeld, Germany) were injected intraperitoneally at 14 and 21 days of age with 30 mg azaserine (Calbiochem-Bering, La Jolla, CA, USA) per kg body weight according to an injection protocol described previously (Woutersen *et al.*, 1989). Ten rats were injected with saline instead of azaserine and served as untreated controls. All animals were housed under similar standard conditions. The rats were killed 15 months after the last injection with azaserine. The animals were anaesthetised with ether, exsanguinated by cannulating the abdominal aorta and then examined for gross pathological changes. The entire pancreas was excised. Grossly visible pancreatic tumours were separated from normal pancreas and portions of both tissues were frozen and stored in liquid nitrogen immediately after dissection, or fixed in 4% buffered formalin and routinely embedded in paraffin wax.

### *Histology*

At three different levels, ten 5 $\mu$m serial sections were prepared from the paraffin embedded pancreata obtained from treated and untreated rats. One section from each level was stained with haematoxylin and eosin (H&E) and examined by light microscopy. Likewise, one part of the liquid nitrogen frozen pancreas was used for preparation of cryostat sections and the adjacent part was used for the molecular biological techniques. The cryostat sections were fixed for 10 min in 4% buffered formalin, stained routinely with H&E and examined by light microscopy (Scherer *et al.*, 1989). Based on the results of the microscopical examinations, directly adjacent parts of the same tissues were selected for further experiments (such as Northern blotting), defined as having either normal, preneoplastic or tumorous histology.

*Immunohistochemistry*

Two different mouse monoclonal antibodies were used to detect EGFR immunoreactivity in paraffin-embedded pancreas sections obtained from all untreated control rats and from more than 20 azaserine-treated rats. At least two sections from every individual rat were incubated. One antibody was a generous gift from Dr WA Dunn, University of Florida, diluted 1:100 and earlier proved to be suitable for immunohistochemical localisation of EGFR in rat tissue (Simms *et al.*, 1991), the other EGFR antibody was purchased from Sigma (St. Louis, MO; clone 29.1), diluted 1:2000. Both antibodies were directed to the extracellular domain of EGFR (Chandler *et al.*, 1985), but are most probably directed against different epitopes, since the antibody from Dr Dunn was reported to interfere with EGF binding to the EGFR, whereas the Sigma antibody does not interfere with this ligand–receptor binding. To demonstrate differences in mitotic rate, the proliferation marker PCNA (proliferating cell nuclear antigen; Santa Cruz Biotechnology, CA, USA; dilution 0.2 $\mu g\ \mu l^{-1}$) was detected on parallel sections. After deparaffinisation, endogenous peroxidase activity was quenched by incubation 0.6% hydrogen peroxide in methanol for 30 min. The sections were pretreated with 0.3% Triton X-100 in phosphate-buffered saline (PBS, 0.14 mM sodium chloride; 8.93 mM disodium hydrogen phosphate; 1.28 mM sodium dihydrogen phosphate; pH 7.4) for 15 min. The slides were incubated for 2 h with the first antibody in 2% bovine serum albumin (BSA) in PBS in a humid slide chamber. To detect the monoclonal antibody, subsequent peroxidase-conjugated rabbit anti-mouse antibody and peroxidase-conjugated swine anti-rabbit antibody (RAMPO/SWARPO; Dako, Glostrup, Denmark; duluted 1:100) incubations were performed. Both RAMPO and SWARPO were diluted in PBS containing 10% normal rat serum and incubated for 30 min. Between each incubation step the sections were washed three times for 5 min with PBS–Tween (0.05%), with the exception of the last washing step where Tween was omitted. Subsequently, a brown precipitate could be observed by light microscopy after the peroxidase reaction with 3,3′-diaminobenzidine tetrahydrochloride (DAB; Sigma). The sections were counterstained with Mayer's haematoxylin. Expression of EGFR in this study is defined as staining of cell membranes, often accompanied by cytoplasmic staining.

*RNA isolation*

Total RNA was isolated by a modification of the procedures described by Chomczynski and Sacchi (1987) and Chirgwin *et al.* (1979). Standard precautions were taken to prevent contamination of solutions and glasswork with RNAases (Sambrook *et al.*, 1989). RNA was isolated from parts of total pancreas and from the grossly visible pancreatic adenocarcinomas isolated at final autopsy. Unfortunately, because of the extremely high RNAase content in rat pancreas, in combination with the relatively long time needed to dissect out the small, but macroscopically visible preneoplastic lesions, it appeared to be impossible to isolate good quality RNA from these small lesions. Therefore, effects on RNA levels in preneoplastic lesions were only detected in total pancreas homogenates containing both 'normal' tissue and lesions. Frozen tissue samples (0.1–0.3 g) were homogenised in a high-speed homogeniser (Ultra Turrax; Janke and Kukel-IKA, Staufen, Germany: 15–20 s at 25 000 r.p.m.) in 3–5 ml of GuSCN (4 M guanidium thiocyanate; 1% β-mercaptoethanol, 0.5% *N*-lauroylsarkosine; 25 mM sodium citrate). RNA was separated from DNA and proteins by ultracentrifugation at 40 000 r.p.m. overnight through a 5.7 M caesium chloride cushion or by acid–phenol–chloroform extraction and subsequent precipitation at −20°C with 0.025 volume 1 M acetic acid and 0.5 volume ethanol. After removing the supernatant, the RNA pellet was redissolved in 800 μl GuSCN. RNA was precipitated again with 0.025 volume 1 M acetic acid and 0.6 volume ethanol. Finally, the RNA pellet was washed in 70% ethanol, dried for 5–10 min in a Speedvac (SVC100H; Savant, Farmingdale, NY, USA), and redissolved in diethylpyrocarbonate (DEPC)-treated water. Quantity and quality of the RNA were monitored by spectrophotometry at 260 nm and 1% agarose gel electrophoresis respectively.

*Northern blotting*

A sample of 25 μg total RNA was denatured in sample buffer (10 × MOPS–37% formaldehyde–formamide = 2:3:10) for 5 min at 65°C, and separated by size on a 1.2% denaturating gel for 4–5 h. Ethidium bromide was added to the RNA sample just before loading of the gel. Equality and integrity of RNA loading was checked by examining ethidium bromide staining intensities of the ribosomal bands. RNA was transferred to a nylon membrane and cross-linked by UV Prehybridisation and hybridisation were performed overnight at 65°C. Typically, $0.5-1 \times 10^6$ c.p.m. $ml^{-1}$ of the $^{32}P$-labelled rat EGFR riboprobe was used for hybridisation in the presence of hybmix (50% formamide; 0.4% sodium dodecyl sulphate(SDS); 4 × SSC; 4 × Denhardt's; 0.2 mg $ml^{-1}$ ssDNA; 0.04 M sodium phosphate pH 7.4; 10% dextran sulphate). Blots were washed twice at low stringency (1 × SSPE; 0.5% SDS; 15 min at 65°C) and once or twice at high stringency (0.1 × SSPE; 0.5% SDS; 15 min at 65°C). The blots were exposed to a phosphor storage screen for 2 days and subsequently scanned by a phosphorimager (Personal Densitometer, Molecular Dynamics, Sunnyvale, CA, USA). A total of 22 pancreatic RNA samples obtained from 17 different rats [four untreated controls (pancreata without any lesions), 12 azaserine-treated rats (pancreata with preneoplastic lesions and five macroscopically isolated acinar adenocarcinomas)] were used for quantification of EGFR mRNA levels. Only RNAs from one isolation blotted on one individual membrane showing equally bright ribosomal bands and equal amount of degradation on the original agarose gel were used for quantitative comparisons. This approach resulted in four independent Northern blots to be used for quantification. EGFR mRNA levels were calculated as percentages of 7S RNA (detected with a 7S-specific DNA probe, Balmain *et al.*, 1982) using the computer program ImageQuant (Molecular Dynamics), and subsequently statistically correlated with the number of lesions observed in parallel sections.

In situ *hybridisation*

Duplicate, 5 μm-thick, formalin-fixed, paraffin-embedded tissue sections from two untreated controls and two azaserine-treated rats were placed on poly-L-lysine (1 mg $ml^{-1}$) coated glass slides, and subsequently deparaffinised, hydrated, permeabilised with 1 μg $ml^{-1}$ proteinase K (Boehringer Mannheim) for 10 min at 37°C, and prehybridised in hybridisation buffer (50% formamide, 20 mM Tris-HCl pH 8.0, 5 mM EDTA, 1 × Denhardt's, 5% dextran sulphate, 10 mM dithiothreitol, 0.3 M sodium chloride) for 4 h at 42°C. As a negative control for mRNA hybridisation, the sections were treated with 200 μg $ml^{-1}$ RNAase A, 10 U $ml^{-1}$ RNAase T1 for 1 h at 37°C just before prehybridisation. As positive controls, hybridisations have been performed with probes directed against insulin. Hybridisation was performed overnight at 50°C with $2.5 \times 10^5$ c.p.m. of $^{33}P$-labelled rat EGFR riboprobe and

1 $\mu$l of tRNA (50 $\mu$g $\mu l^{-1}$) in 200 $\mu$l of hybridisation buffer per slide. After hybridisation, non-bound probe was digested with RNAase A (Boehringer Mannheim, 20 $\mu$g $\mu l^{-1}$) for 30 min at 37°C, and aspecific binding was washed with increasing stringency. The sections were coated with autoradiography emulsion (Eastman Kodak, Rochester, NY, USA) and exposed for 5–10 days. The sections were developed and fixed and subsequently counterstained with haematoxylin.

*Preparation of the rat EGFR riboprobe*

The rat EGF receptor cDNA fragment, corresponding to nucleotide bases 249–951 (Petch *et al*., 1990) was amplified by PCR from reverse-transcribed rat liver RNA using PCR primers which contained unique *Bam*HI and *Sph*I restriction sites. The PCR products were generated in 40 cycles (94°C, 1.5 min; 42°C, 1.5 min; and 72°C, 1.5 min), subsequently subcloned into a pGEM7Zf vector (Promega), and authenticity was confirmed by sequencing.

### Results

*Immunohistochemistry*

In the normal rat pancreas, immunohistochemical studies with monoclonal antibodies directed to the EGFR demonstrated expression in the cytoplasm of acinar cells, sometimes with accentuation of the cell membranes (Figure 1). Both anti-EGFR monoclonal antibodies gave similar staining patterns in all tissue sections that were investigated. Pancreatic cells of rats not treated with azaserine showed similar staining patterns to histologically normal pancreatic cells of rats that were treated with azaserine. No differences in EGFR staining intensities were observed in non-pancreatic cells (such as vascular endothelial cells, macrophages or nerve fibres) when comparing azaserine-treated with untreated rats. Ductular and endocrine cells were negative. Surprisingly, normal acinar cells stained more intensely for EGFR than the eosinophilic acinar cells of putative preneoplastic atypical acinar cell lesions (AACN; Figures 1 and 2). More advanced lesions such as nodules-in-nodules (Figure 3a), carcinomas *in situ* and acinar adenocarcinomas were negative. The AACN exhibited a large variation in their intensity of EGFR immunoreactivity (Figure 2), which was found predominantly in the cytoplasm in these lesions since hardly any immunoreactivity to EGFR was present on the cellular membranes. The AACN also varied in number of cells with immunoreactivity to EGFR. In some lesions only a few dispersed cells were positive, whereas other lesions showed an almost similar immunoreactivity to EGFR as the surrounding normal acinar cells. Figure 2 demonstrates clearly the variance in EGFR immunoreactivity within one pancreas tissue section, ranging from hardly any staining through a staining intensity similar to normal acinar cells. In general, larger (more advanced) atypical acinar cell lesions, when

**Figure 1** Immunohistochemical localisation of EGFR in azaserine-treated rat pancreas. I, islet of Langerhans cells; A, normal pancreas acinar cells; P, putative preneoplastic atypical acinar cell nodule; arrowheads point to membranous staining. The antibody used is MAb 29.1 (Sigma); bar = 50 $\mu$m.

**Figure 2** Immunohistochemical localisation of EGFR in azaserine-treated rat pancreas. Note differences in intensities of EGFR in the putative preneoplastic lesions. P, putative preneoplastic atypical acinar cell nodule. The antibody used is MAb 29.1 (Sigma); bar = 400 $\mu$m.

**Figure 3** Immunohistochemical localisation of (**a**) EGFR and (**b**) PCNA, in azaserine-treated rat pancreas. A, normal pancreas acinar cells; P, putative preneoplastic atypical acinar cell nodule (primary lesion); N, nodule-in-nodule (secondary lesion). The antibody used is MAb 29.1 (Sigma); bar = 200 $\mu$m.

loosing their typical nodular (circular) shape, also lost their EGFR immunoreactivity. However, no obvious variations in staining patterns could be observed between the pancreatic sections of different rats.

Immunohistochemical detection of the proliferation marker PCNA in parallel sections clearly demonstrated an inverse relationship between mitotic rate and immunoreactivity to EGFR (Figure 3b). In contrast with the putative preneoplastic eosinophilic atypical acinar cell foci, the basophilic atypical acinar cell foci (which are not considered to be preneoplastic) could not be distinguished from normal acinar cells by incubation with either the anti-EGFR antibodies or the PCNA antibody (not shown). Sections of the rat submaxillary and sublingual salivary glands were used as controls for the specificity of the anti-EGFR antibodies. As expected, strong immunoreactivity was apparent only on the serous cells. Omitting the anti-EGFR or anti-PCNA first antibodies resulted in complete absence of staining.

*Northern blotting*

A total of 17 rat pancreatic tissues comprising normal and putative preneoplastic acinar cells, and five macroscopically isolated acinar adenocarcinomas were analysed by Northern blotting (Figure 4). No differences in EGFR mRNA expression were observed in homogenates from untreated controls or from rats treated with azaserine with no, or a very low number of AACN in their pancreas (observed in parallel sections from the pancreatic pieces used for homogenisation). A significantly positive correlation was found for the EGFR mRNA levels when compared with the number of atypical acinar cell lesions found in parallel sections from the same pancreata (four independent Northern blots: $r=0.9950$, $P<0.05$; $r=0.9993$, $P<0.01$; $r=0.85263$; $P<0.05$; $r=0.9953$, $P<0.05$). However, no EGFR mRNA expression was detected by Northern blotting on total RNA from the isolated acinar adenocarcinomas, whereas ethidium bromide staining intensity and 7S control signal were equally detectable (Figure 4).

**Figure 4** EGFR mRNA levels detected by Northern blotting in pancreatic homogenates from azaserine-treated rats. The blots were rehybridised with a 7S cDNA probe in order to quantify RNA loading. Lane 1, normal pancreas; lane 2, preneoplastic pancreas; lane 3, acinar adenocarcinoma. 28S, 18S, location of the ribosomal subunits.

In situ *hybridisation*

Hybridisation of rat pancreas sections with the $^{33}P$-labelled rat EGFR riboprobe revealed a positive signal in the acinar cells of normal pancreas, whereas no grains could be observed in the ductular (Figure 5a, exposure time 10 days) and endocrine cells (not shown). Furthermore, putative preneoplastic atypical acinar cell nodules exhibited a similar number or even more grains than the normal acinar cells (Figure 5b, exposure time 5 days). However, in more advanced lesions (e.g. carcinoma *in situ*), EGFR mRNA was only faintly detectable (Figure 5c, exposure time 10 days) or undetectable. When the sections were pretreated with RNAase, no differences in signals were observed between normal, preneoplastic or neoplastic tissues; the few remaining grains were considered to represent background (Figure 5d, exposure time 10 days).

### Discussion

Using immunohistochemistry, Northern blotting, and *in situ* hybridisation, the present study clearly demonstrates that epidermal growth factor receptors (EGFRs) are detectable on pancreatic acinar cells of normal rats and of rats treated with azaserine, as summarised in Table I. By Northern blotting, the presence of a high number of putative preneoplastic atypical acinar cell nodules (AACN) was associated with increased expression of EGFR mRNA compared with pancreatic tissues containing no or a low number of AACN. In contrast, by immunohistochemistry, most AACN exhibited decreased EGFR immunoreactivity, indicating a decrease in the amount of EGFR protein. The discordance between EGFR mRNA and protein levels could be due to inhibition of EGFR at the translational level, increased EGFR protein degradation or shedding of EGFR by the AACN cells. Another explanation could be that up-regulation of EGFR in putative preneoplastic acinar pancreatic cells of rats contributes to cell proliferation, but that a high degree of receptor signalling and turnover within the lesion results in a relatively low level of observable receptor protein at any point of time.

EGFR was not detectable in acinar adenocarcinomas by either immunohistochemistry, *in situ* hybridisation or Northern blotting, indicating that in advanced acinar lesions, transcription of DNA coding for EGFR either does not take place or EGFR mRNA is markedly and rapidly degraded. These findings suggest that EGFR may not play an essential role in the pancreatic carcinogenic process in azaserine-treated rats. Alternatively, EGFR may be involved in the regulation of differentiated functions in the rat pancreatic acinar cell and may exert growth-suppressive effects on this cell type.

It cannot be excluded that diminished EGFR immunoreactivity as observed in the present study is the result of a change of the epitope instead of actual decrease in the number of EGFR present. However, this explanation does not seem to be very likely, since we found similar results with two different monoclonal antibodies directed to the EGFR. Moreover, it has been established that the antibodies used are reactive in rats since they demonstrate a positive reaction with salivary glands collected from the same animals.

It is conceivable that the findings presented in this paper are typical for acinar pancreatic cells only and may very well not apply for ductular pancreatic cells or ductular adenocarcinomas of the pancreas. To our knowledge, human acinar adenocarcinomas have not been investigated for the presence of EGFR. Similar experiments to those described in this paper are performed in our Institute with *N*-nitrosobis(2-oxopropyl)amine-treated hamsters as a model for ductular adenocarcinomas (Pour and Wilson, 1980), in order to investigate whether EGFR might play a role in the development of pancreatic ductular carcinomas.

Decreased EGFR-mediated signalling may thus lead to

**Figure 5** EGFR mRNA detection by *in situ* hybridisation in azaserine-treated rat pancreas. (**a**) A, acinar cells; D, ductular cells. (**b**) A, acinar cells; P, putative preneoplastic atypical acinar cell nodule. (**c**) A, normal pancreas acinar cells; C, adenocarcinoma. (**d**) after RNAase treatment; A, normal pancreas acinar cells; P, putative preneoplastic atypical acinar cell nodule. Differences in signal intensities between sections are due to differences in exposure times; bar = 50 μm.

**Table I** Summary of the results obtained from pancreatic acinar cells of azaserine-treated rats[a]

| *Phenotype* | *EGFR protein (IHC)* | *EGFR mRNA (ISH, North.)* | *PCNA (IHC)* |
|---|---|---|---|
| Normal | + | + | ± |
| Putative preneoplastic | ± | +/++ | ±/+/++ |
| Neoplastic | − | − | ++ |

[a]The values are based on microscopic examinations of pancreatic acinar cells or densitometrical data as indicated in the text. Values are relative levels in the different tissue types: −, not present; ±, moderately present; +, clearly present; ++, strongly present; IHC, immunohistochemistry; ISH, *in situ* hybridisation; North., Northern blotting; EH, enzyme histochemistry.

loss of differentiated functions and increased propensity toward neoplastic transformation. Indeed, several lines of evidence suggest an anti-mitogenic role for EGFR in the rat exocrine pancreas. First, the rat pancreatic acinar cell has specific high-affinity EGF receptors (Korc *et al.*, 1983), and EGF is necessary for the maintainance of this cell type in serum-free culture (Brannon *et al.*, 1985). Second, EGF enhances rat acinar cell survival and pancreatic protein synthesis at concentrations as low as 42 pM (Brannon *et al.*, 1985; 1986; 1988). Third, this action of EGF is relatively specific, inasmuch as a similar effect occurs only at 2.7 nM IGF-1 and does not occur with insulin (Brannon *et al.*, 1988). Fourth, EGF decreases thymidine incorporation into pancreatic DNA in male, Sprague–Dawley rats (Morisset *et al.*, 1989) and increases pancreatic content of amylase and chymotrypsinogen while preventing caerulein-mediated desensitisation of the acinar cell secretory responsiveness (Morisset *et al.*, 1989). Fifth, EGF binding is decreased in the regenerating rat pancreas following 90% pancreatectomy, in parallel with an increase in acinar cell mitotic activity (Brockenbrough *et al.*, 1988). Sixth, the results of a recent study performed by our group demonstrated that putative preneoplastic atypical acinar cell lesions may develop into acinar adenocarcinomas independently of TGF-α or EGF (Visser *et al.*, 1995). Thus, a decrease in EGFR-mediated signalling in the rat pancreatic acinar cell may lead to enhanced carcinogenesis.

However, another explanation could be that cells within the neoplastic lesion may have accumulated other genetic alterations e.g. in other members of the EGFR family, such as c-*erb*B-2 (HER-2, ERBB2, *neu*; Yamamoto *et al.*, 1986; Coussens *et al.*, 1985; Stern *et al.*, 1986) or ERBB3 (Kraus *et al.*, 1989; Plowman *et al.*, 1990), driving cell division without the necessity for EGFR signalling. Consequently, the EGFR gene is generally switched off in the malignant adenocarcinoma cells.

**Abbreviations**

AACN, atypical acinar cell nodules; EGF(R), epidermal growth factor (receptor); ISH, *in situ* hybridisation; mRNA, messenger RNA; PCNA, proliferating cell nuclear antigen; RAMPO, peroxidase-conjugated rabbit–anti-mouse antibody; SWARP, peroxidase-conjugated swine anti-rabbit antibody.

**Acknowledgements**

The authors would like to thank Dr WA Dunn, University of Florida, for his kind gift of a monoclonal antibody directed to the EGFR. The authors are grateful to Professors VJ Feron and JG van den Tweel for critical evaluation of the manuscript. This work was supported by a grant from the Dutch Cancer Society, CIVO 91-01, and by Public Health Service Grant CA-40162, awarded by the National Cancer Institute, USA.

### References

BALMAIN A, KRUMLAUF R, VASS JK AND BIRNIE GD. (1982). Cloning and characterization of the abundant cytoplasmic 7S RMA from mouse cells. *Nucleic Acids Res.*, **10**, 4259–4277.

BRANNON PM, ORRISON BM AND KRETCHMER N. (1985). Primary culture of rat pancreatic acinar cells in serum-free medium. *In vitro Cell. Dev. Biol.*, **21**, 6–14.

BRANNON PM, DEMAREST AS, SABB TE AND KORC M. (1986). Dietary modulation of epidermal growth factor action in cultured pancreatic acinar cells. *J. Nutr.*, **116**, 1306–1315.

BRANNON PM, HIRSCHI K AND KORC M. (1988). Effects of epidermal growth factor, insulin, and insulin-like growth factor on rat pancreatic acinar cells cultured in serum-free medium. *Pancreas*, **3**, 41–48.

BROCKENBROUGH JS, WEIR GC AND KORC M. (1988). Alterations in EGF binding to acini during pancreatic regeneration in the rat. *Int. J. Pancreat.*, **3**, 415–424.

CHANDLER LP, CHANDLER CE, HOSANG M AND SHOOTER EM. (1985). A monoclonal antibody which inhibitis epidermal growth binding has opposite effects on the biological action of epidermal growth factor in different cells. *J. Biol. Chem.*, **260**, 3360–3367.

CHIRGWIN JM, PRZYBYLA AE, MCDONALD RJ AND RUTTER WJ. (1979). Isolation of biologically active ribonucleic acid from sources enriched of ribonuclease. *Biochemistry*, **18**, 5294–5299.

CHOMCZYNSKI P AND SACCHI N. (1987). Single-step method of RNA isolation by acid guanidium thiocyanate–phenol–chlorophorm extraction. *Anal. Biochem.*, **162**, 156–159.

COUSSENS L, YANG-FENG TL, LIAO Y-C, CHEN E, GRAY A, MCGRATH J, SEEBURG PH, LIBERMANN TA, SCHLESSINGER J, FRANCKE U, LEVINSON A AND ULLRICH A. (1985). Tyrosine kinase receptor with extensive homology to EGF receptor shares chromosomal location with neu oncogene. *Science*, **230**, 1132–1139.

DOWNWARD J, YARDEN Y, MAYES E, SCARCE G, TOTTY N, STOCKWELL P, ULLRICH A, SCHLESSINGER J AND WATERFIELD MD. (1984). Close similarity of epidermal growth factor receptor and v-*erb*B oncogene protein sequences. *Nature*, **307**, 521–527.

ELDER JT. (1994). Transforming growth factor-α and related growth factors. In *Epidermal Growth Factors and Cytokines*, Luger TA and Schwarz T. (eds) pp. 205–240. Marcel Dekker: New York.

GAMOU SK, KIM YS AND SHIMIZU N. (1984). Different responses to EGF in two human carcinoma cell lines, A431 and UCVA-1, possessing high numbers of EGF receptors. *Mol. Cell. Endocrinol.*, **37**, 205–213.

KORC M, MATRISIAN ML, PLANCK SR AND MAGUN BM. (1983). Binding of epidermal growth factor in rat pancreatic acini. *Biochem. Biophys. Res. Commun.*, **111**, 1066–1073.

KORC M, MELTZER P AND TRENT J. (1986). Enhanced expression of epidermal growth factor receptor correlates with alterations of chromosome 7 in human pancreatic cancer. *Proc. Natl Acad. Sci. USA*, **83**, 5141–5144.

KORC M, CHANDRASEKAR B, YAMANAKA Y, FRIESS H, BUCHLER M AND BEGER HG. (1992). Overexpression of the epidermal growth factor receptor in human pancreatic cancer is associated with concomitant increases in the levels of epidermal growth factor and transforming growth factor alpha. *J. Clin. Invest.*, **90**, 1352–1360.

KRAUS MH, ISSING W, MIKI T, POPESCU NC AND AARONSON SA. (1989). Isolation and characterization of ERBB3, a third member of the ERBB/epidermal growth factor receptor family: evidence for overexpression in a subset of human mammary tumours. *Proc. Natl Acad. Sci. USA*, **86**, 9193–9197.

LANG RA AND BURGESS AW. (1990). Autocrine growth factors and tumorigenic transformation. *Immunol. Today*, **11**, 244–249.

LEMOINE NR, HUGHES CM, BARTON CM, POULSOM R, JEFFERY RE, KLÖPPEL G, HALL PA AND GULLICK WJ. (1992). The epidermal growth factor receptor in human pancreatic cancer. *J. Pathol.*, **166**, 7–12.

LONGNECKER DS. (1983). Early morphologic markers of carcinogenicity in rat pancreas. In *Application of Biological Markers to Carcinogen Testing*, Milham HA and Sells S. (eds) pp. 43–60. Plenum Press: New York.

MERLINO GT, XU Y-H, ISHII S, CLARK AJL, SEMBA K, TOYOSHIMA K, YAMAMOTO T AND PASTAN I. (1984). Amplification and enhanced expression of the epidermal growth factor receptor gene in A431 human carcinoma cells. *Science*, **224**, 117–119.

MORISSET J, LAROSE L AND KORC M. (1989). *In vivo* effects of epidermal growth factor and caerulein on acinar cell function in rat pancreas. *Endocrinology*, **124**, 2693–2698.

PAS MFW TE, BERGEN EN, HENEGOUWEN PMP VAN, BOONSTRA J AND PONEC M. (1991). Regulation of epidermal growth factor receptor expression in normal and transformed keratinocytes. *Arch. Dermatol. Res.*, **283**, 125–130.

PETCH LA, HARRIS J, RAYMOND VW, BLASBAND A, LEE DC AND EARP HS. (1990). A truncated, secreted form of the epidermal growth factor receptor is encoded by an alternatively spliced transcript in normal rat tissue. *Mol. Cell. Biol.*, **10**, 2973–2982.

PLOWMAN GD, WHITNEY GS, NEUBAUER MG, GREEN JM, MCDONALD VL, TODARO GJ AND SHOYAB M. (1990). Molecular cloning and expression of an additional epidermal growth factor receptor-related gene. *Proc. Natl Acad. Sci. USA*, **87**, 4905–4909.

POUR PM AND WILSON RB. (1980). Experimental tumors in the pancreas. In *Tumors of the Pancreas*, Moossa AR. (ed.) pp. 37–158. Williams and Wilkins: Baltimore.

SAMBROOK J, FRITSCH EF AND MANIATIS T. (1989). *Molecular Cloning: a Laboratory Manual*, 2nd ed., Cold Spring Harbor Laboratory Press: New York.

SCHERER E, BAX J AND WOUTERSEN RA. (1989). Pathogenic interrelationship of focal lesions, nodules, adenomas and carcinomas in the multistage evolution of azaserine-induced rat pancreas carcinogenesis. In *Biologically Based Methods for Cancer Risk Assessment*, NATO ASI Series, 159, Travis CC. (ed.) pp. 41–54. Plenum Press: New York and London.

SIMMS JS, CHEGINI N, WILLIAMS RS, ROSSI AM AND DUNN WA. (1991). Identification of epidermal growth factor, transforming growth factor-alpha, and epidermal growth factor receptor in surgically induced endometriosis in rats. *Obstet. Gynecol.*, **78**, 850–857.

SMITH JJ, DERYNCK R AND KORC M. (1987). Production of transforming growth factor-α in human pancreatic cancer cells: evidence for a superagonistic autocrine cycle. *Proc. Natl Acad. Sci. USA*, **84**, 7567–7570.

STERN DF, HEFFERNAN PA AND WEINBERG RA. (1986). p185, a product of the neu proto-oncogene, is a receptorlike protein associated with tyrosine kinase activity. *Mol. Cell. Biol.*, **6**, 1729–1740.

TEMIN HM. (1966). Studies on carcinogenesis by avian sarcoma viruses. 3. The differential effect of serum and polyanions on multiplication of uninfected and converted cells. *J. Natl Cancer Inst.*, **37**, 167–175.

VELU TJ. (1990). Structure, function and transforming potential of the epidermal growth factor receptor. *Mol. Cell. Endocrinol.*, **70**, 205–216.

VISSER CJT, WOUTERSEN RA, BRUGGINK AH, GARDEREN-HOETMER A VAN, SEIFERT-BOCK I, TILANUS MGJ AND DE WEGER RA. (1995). Transforming growth factor-α and epidermal growth factor expression in the exocrine pancreas of azaserine-treated rats: modulation by cholecystokinin or a low fat, high fiber (caloric restricted) diet. *Carcinogenesis*, **16**, 2075–2082.

WARSHAW AL AND CASTILLO CF DEL. (1992). Pancreatic carcinoma. Review article. *N. Engl. J. Med.*, **326**, 455–465.

WOUTERSEN RA, GARDEREN-HOETMER A VAN, BAX J AND SCHERER E. (1989). Modulation of dietary fat-promoted pancreatic carcinogenesis in rats and hamsters by chronic ethanol ingestion. *Carcinogenesis*, **10**, 453–459.

YAMAMOTO T, IKAWA S, AKIYAMA T, SEMBA K, NOMURA N, MIYAJIMA N, SAITO T AND TOYOSHIMA K. (1986). Similarity of protein encoded by the human c-*erb*B-2 gene to epidermal growth factor receptor. *Nature*, **319**, 230–234.